# Supplementary material for: Deconvolution to restore cryo-EM maps with anisotropic resolution
Source: bioRxiv. 2025 Mar 1:2025.02.23.639707. Preprint. [Version 1] doi: 10.1101/2025.02.23.639707 (PMC11888254; doi:10.1101/2025.02.23.639707)
Supplement: Supplement 1 [file NIHPP2025.02.23.639707v1-supplement-1.pdf]

bioRxiv preprint doi: https://doi.org/10.1101/2025.02.23.639707; this version posted March 1, 2025. The copyright holder for this preprint (which was not certified by peer review) is the author/funder, who has granted bioRxiv a license to display the preprint in perpetuity. It is made available under aCC-BY 4.0 International license.

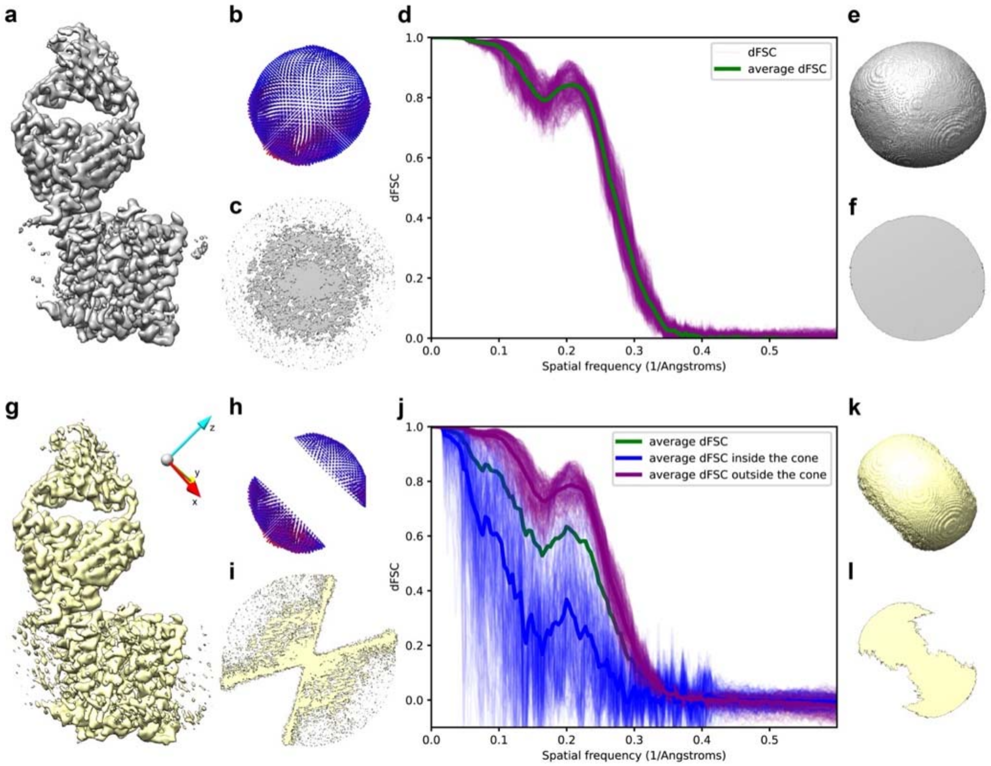


**Supplementary Figure 1. Characterizations of datasets with and without preferred orientation.** **a**, Ground truth reconstruction from the original dataset with uniform angular distribution. **b**, Angular distribution of the original datasets. **c**, Central z-slices through 3D Fourier transforms of map in (**a**). **d**, dFSC curves calculated between two independent half-maps refined from particles with uniform angular distribution. The thick green line represents the average dFSC, while the thin purple lines show individual dFSC curves for all directions. **e** and **f**, The 3D dFSC volume (**e**) rendered from (**d**) and its central z-slice along z-axis (**f**), demonstrating isotropic resolution. **g**, Distorted reconstruction from a synthetic dataset with preferred orientation along the z-axis. **h**, Angular distribution of z-axis preferred orientation datasets. **i**, Central z-slices through 3D Fourier transforms of the distorted map shown in (**g**). **j**, dFSC curves calculated between two independent half-maps refined from particles with z-axis preferred orientation. Thin blue lines are dFSC curves inside the cones. **k** and **l**, 3D dFSC volume (**k**) derived from (**j**) and its z-slice (**l**), revealing missing information and reduced resolution specifically along z-axis, the axis of preferred orientation.

bioRxiv preprint doi: https://doi.org/10.1101/2025.02.23.639707; this version posted March 1, 2025. The copyright holder for this preprint (which was not certified by peer review) is the author/funder, who has granted bioRxiv a license to display the preprint in perpetuity. It is made available under aCC-BY 4.0 International license.

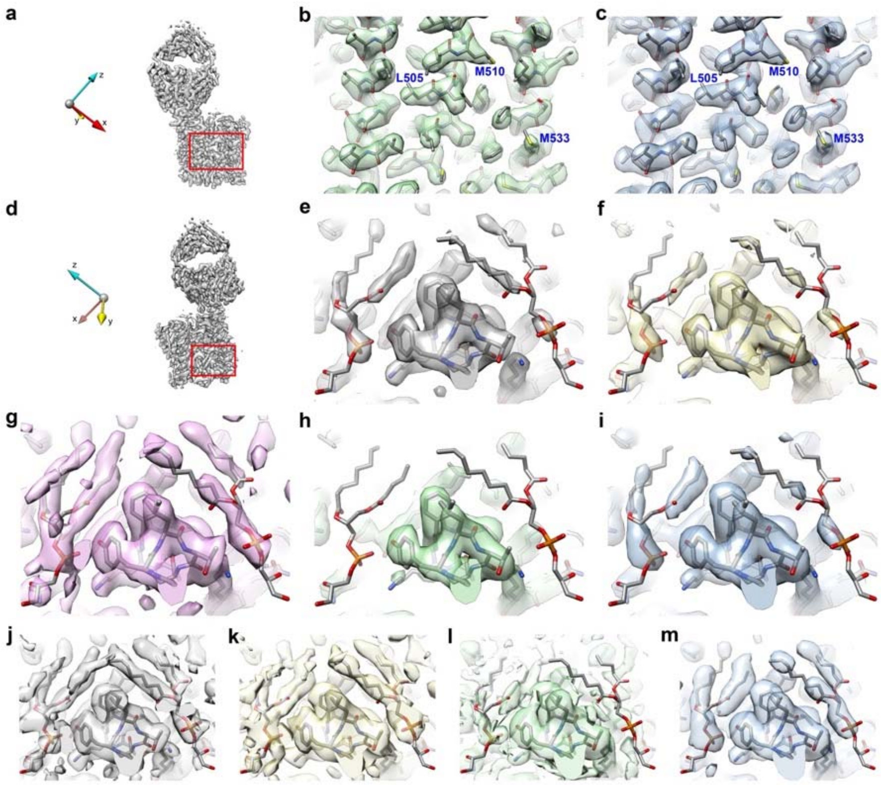


**Supplementary Figure 2. Comparison of maps processed in different ways.**
**a**, The red box indicates the area expanded in panels (**b**) and (**c**). **b** and **c**, Close-up views of maps after processing using DeepEMhancer tightTarget model (**b**) and wideTarget model (**c**), showing reduced elongation of backbone density but no improvement in weakened density (blue labels). **d**, Overview of the ground truth ferroportin map from alternative perspective, focusing on lipids density. The red box highlights the area detailed in panels **e-m**. **e**, Magnified view showing lipids in the ground truth map. **f**, Zoomed view of the map with preferred orientation along z-axis, revealing disrupted lipids density. **g**, Lipid shape is well resolved, and continuity is improved after deconvolution. **h**, Lipid density absent in the map processed using DeepEMhancer tightTarget model. **i**, Lipid density discontinuous in the map processed with DeepEMhancer wideTarget model. **j, k, l** and **m**, Lipids density displayed at lower contour level for maps in (**e**), (**f**), (**h**), (**i**), respectively.

bioRxiv preprint doi: https://doi.org/10.1101/2025.02.23.639707; this version posted March 1, 2025. The copyright holder for this preprint (which was not certified by peer review) is the author/funder, who has granted bioRxiv a license to display the preprint in perpetuity. It is made available under aCC-BY 4.0 International license.

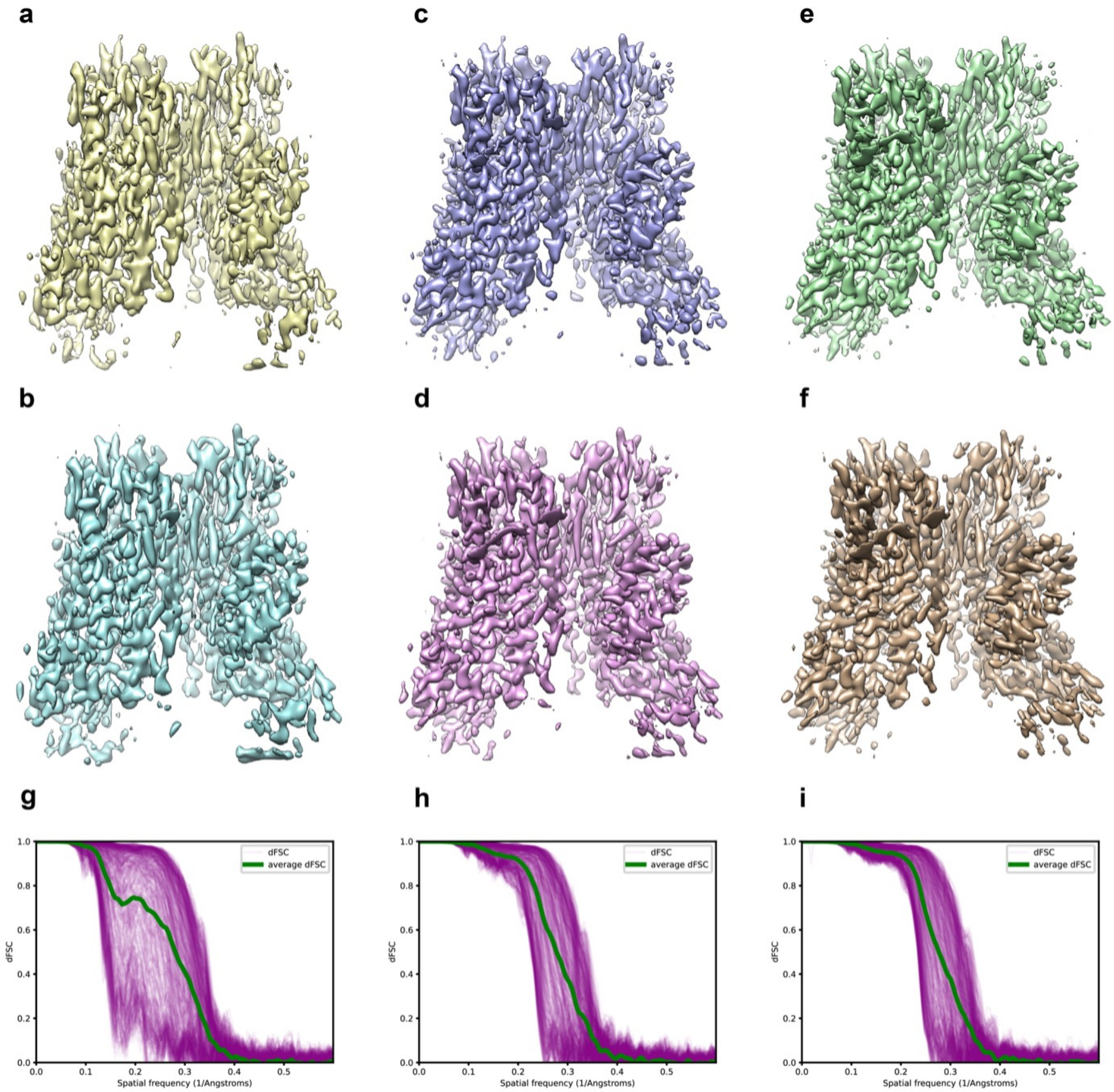


**Supplementary Figure 3 | Results of iterative refinement and deconvolution.**
**a**, Overview of TMEM16A map refined using a raw particle set with strong preferred orientation. **b**, The TMEM16A map after deconvolution. **c**, Refined map using the deconvolved map from (**b**) as a reference. **d**, Further deconvolution of the map in (**c**). **e,f**, Maps after additional round of refinement (**e**) followed by deconvolution (**f**). **g-i**, directional Fourier Shell Correlation (dFSC) curves calculated with a mask using two half-maps from the initial (**g**), second (**h**) and final (**i**) rounds of refinement. The thin purple lines depict dFSC curves calculated along 500 uniformly sampled orientations. The thick green line shows the isotropically averaged dFSC.

bioRxiv preprint doi: https://doi.org/10.1101/2025.02.23.639707; this version posted March 1, 2025. The copyright holder for this preprint (which was not certified by peer review) is the author/funder, who has granted bioRxiv a license to display the preprint in perpetuity. It is made available under aCC-BY 4.0 International license.

**Supplementary Figure 4**. **Deconvolution of TSHR complexes.**

**a**, Top view of a cryo-EM map of the TSH-bound TSHR-Gs complex. **b**, The same view of the map in (**a**) after deconvolution. Deconvolution enhances visualization of secondary structures. **c**, Top view of a cryo-EM map of the TSHR-Gs bound to TR1402, a high affinity analogue of TSH. **d**, The same view of the map in (**c**) after deconvolution. Note that the secondary structures resolved in (**b**) correspond well with those in (**d**).

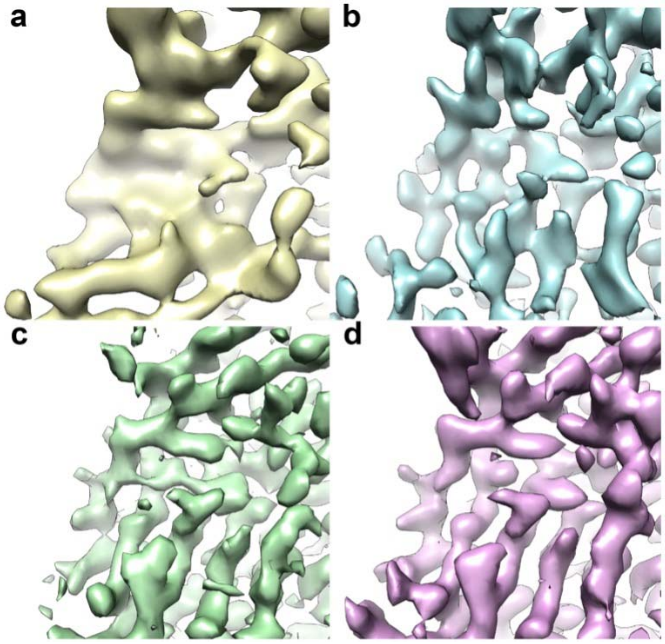

bioRxiv preprint doi: https://doi.org/10.1101/2025.02.23.639707; this version posted March 1, 2025. The copyright holder for this preprint (which was not certified by peer review) is the author/funder, who has granted bioRxiv a license to display the preprint in perpetuity. It is made available under aCC-BY 4.0 International license.

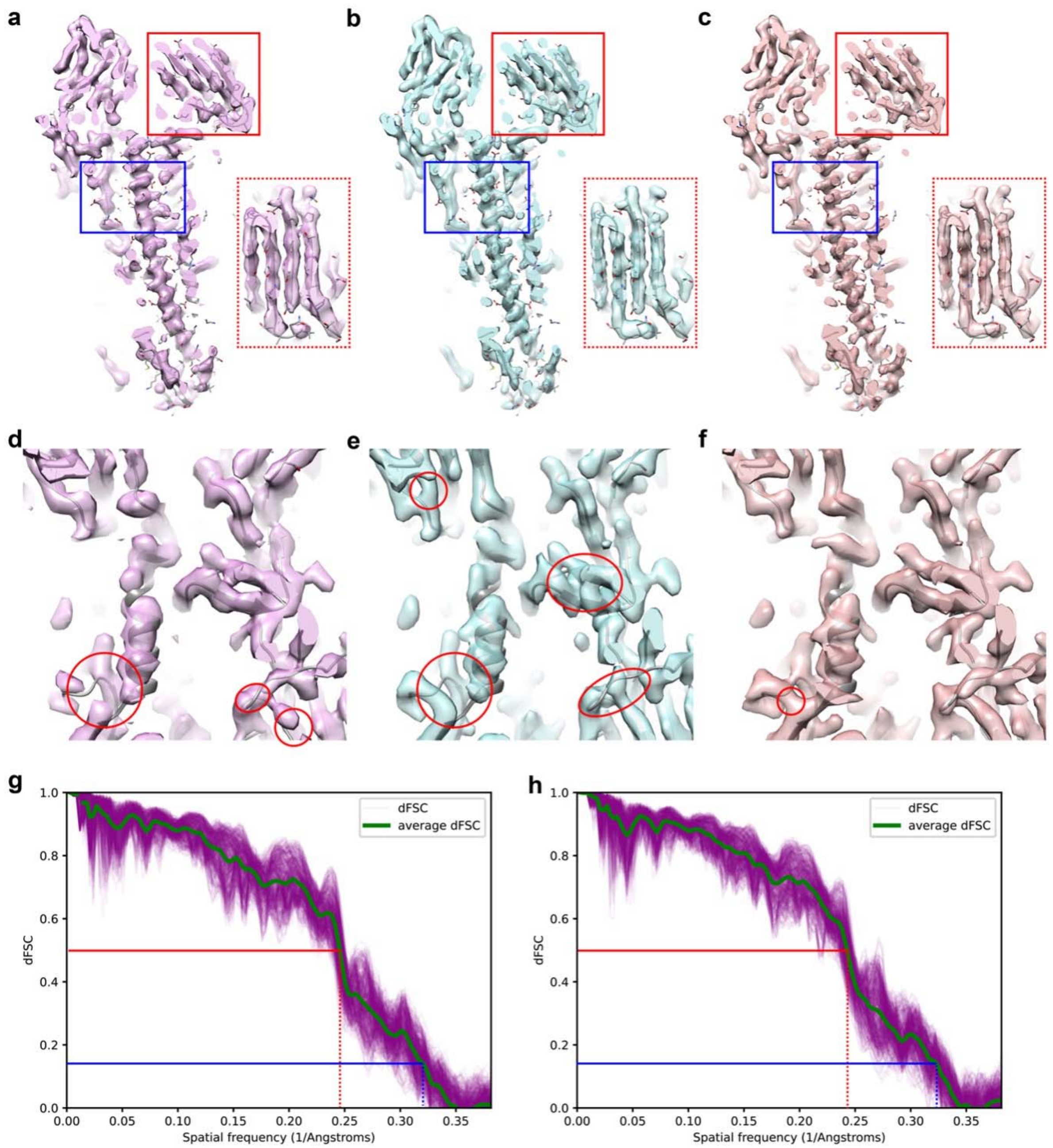


**Supplementary Figure 5. Combination of deconvolution and EMReady**.

**a-c**, Central vertical slices of the HA trimer density map after processing with deconvolution (**a**) and EMReady (**b**) alone, and the same map processed by deconvolution followed by EMReady (**c**) leveraging benefits of both approaches in resolving higher structure details. The blue and red boxes highlight differences between the three maps, with the red dashed boxes indicating enlarged views of the regions marked by the red boxes. **d-f**, Lower half of the HA trimer density map shown in panels (**a-c**), respectively, with red elliptical circles highlighting regions where the backbone density deviates from the atomic mode. **g** and **h**, Map to the ground truth map (emd-21954) dFSC for the HA trimer map after processing with EMReady (**g**) and with the combination of deconvolution and EMReady (**h**).

bioRxiv preprint doi: https://doi.org/10.1101/2025.02.23.639707; this version posted March 1, 2025. The copyright holder for this preprint (which was not certified by peer review) is the author/funder, who has granted bioRxiv a license to display the preprint in perpetuity. It is made available under aCC-BY 4.0 International license.

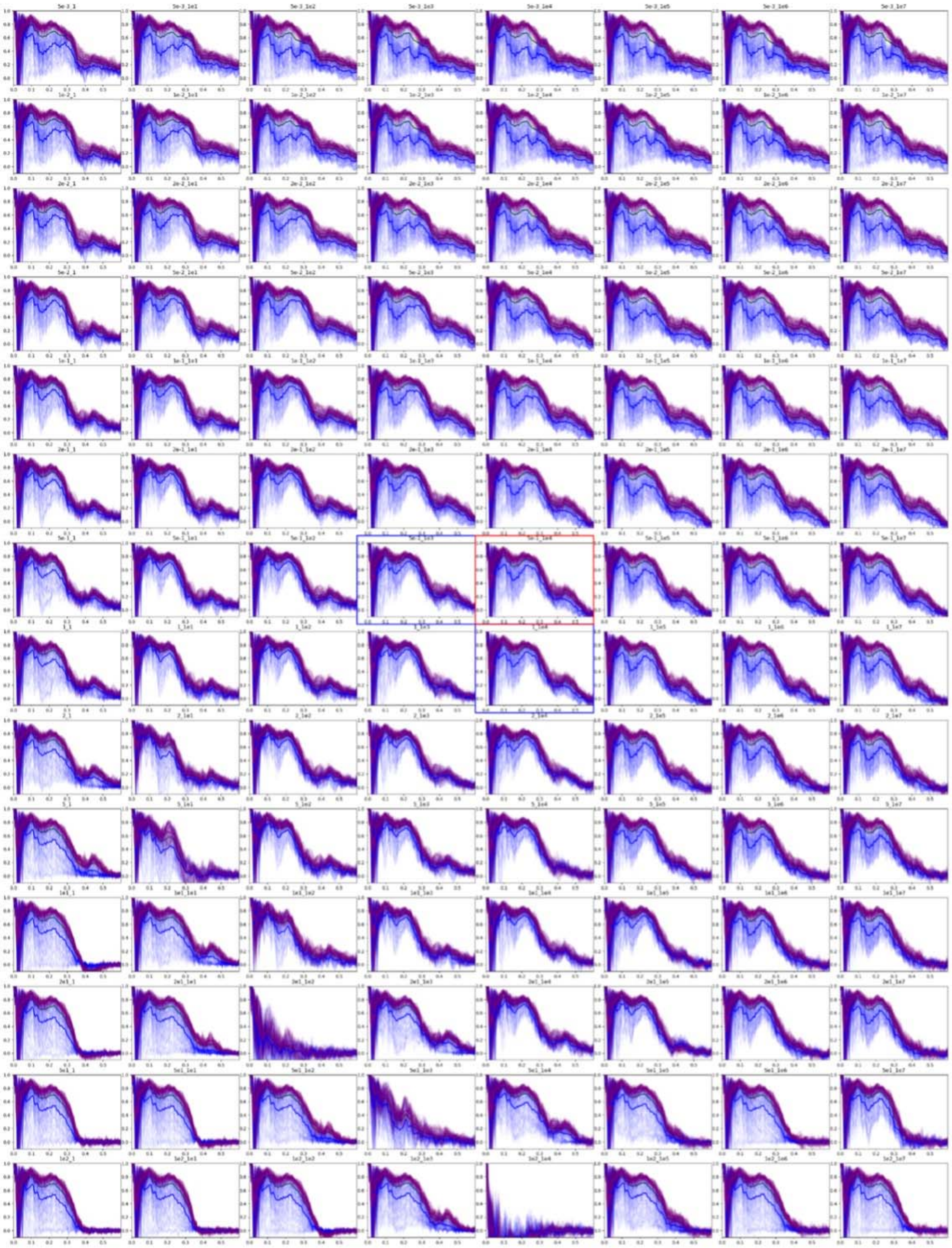

**Supplementary Figure 6. Optimization of smoothness and nonlinearity of ER-Decon.** Optimization and nonlinearity are two major parameters that influences the performance of ER-Decon, thus the performance of AR-Decon. Synthetic Ferroportin dataset was used to screen for optimized permeameters of smoothness and nonlinarity Directional FSC (dFSC).
